# Damage Detection in Active Suspension Bridges: An Experimental Investigation

**DOI:** 10.3390/s18093002

**Published:** 2018-09-07

**Authors:** Fanhao Meng, Bilal Mokrani, David Alaluf, Jingjun Yu, André Preumont

**Affiliations:** 1 Robotics Institute, Beihang University, 100191 Beijing, China; 2 Active Structures Laboratory, Université Libre de Bruxelles, 1050 Brussels, Belgium; David.Alaluf@esa.int (D.A.) andre.preumont@ulb.ac.be (A.P.); 3 Department of Mechanical, Materials and Aerospace Engineering, University of Liverpool, Liverpool L69 3BX, UK; Bilal.Mokrani@liverpool.ac.uk; 4 Optoelectronics Section, ESA-ESTEC, 2201 AZ Noordwijk, The Netherlands

**Keywords:** damage detection, active cable, Hilbert–Huang transform, suspension bridges, health monitoring

## Abstract

This paper considers a Hilbert marginal spectrum-based approach to health monitoring of active suspension bridge hangers. The paper proposes to takes advantage of the presence of active cables and use them as an excitation mean of the bridge, while they are used for active damping. The Hilbert–Huang transform is used to calculate the Hilbert marginal spectrum and establish a damage index for each hanger of the suspension bridge. The paper aims to investigate the method experimentally, through a series of damage scenarios, on a laboratory suspension bridge mock-up equipped with four active cables; each active cable is made of a displacement actuator collocated with a force sensor. Different locations and levels of damage severity are implemented. For the first time, the investigation demonstrates experimentally the effectiveness of the technique, as well as its limitations, to detect and locate the damage in hangers of a suspension bridge.

## 1. Introduction

Over recent years, the improvement in construction materials, construction technology, computing ability, and above all the understanding of the physics of the complex phenomena which control the external loads acting on structures have revolutionized the civil engineering community, enabling the construction of large and elegant civil infrastructures. With the rising of large-scale civil engineering structures, structural health monitoring (SHM) problems have become a crucial scientific issue over the last two decades [1,2,3]. Suspension bridges are among civil structures vulnerable to damages, such as corrosion in the hangers; this study is concerned with this problem.

The goal of health monitoring techniques is to be able to detect, locate and assess the extent of a damage to a structure to predict its safety and lifetime. As an alternative to existing local detection methods, vibration-based methods have been widely applied over the last years [4,5,6,7,8], where most of the damage information could be obtained by analyzing vibration data [9,10,11,12,13]. In general, the vibration data contains two parts, one is the measurable input applied to the bridge system and the other one is the output. From these vibration measurements, the real model of the structure can be obtained by a variety of parameter estimation methods [14]. However, in practice, applying an artificial force as the excitation for the bridge require extensive instrumentation and disruption of traffic which make frequent tests less favorable [15]. For online health monitoring in real suspension bridge, the principal method to excite the bridge is relying upon available ambient excitation sources. It is practically impossible to measure this ambient excitation and the outputs are the only information that can be used [16,17]. In addition, some novel integrated technologies have developed quickly and achieved positive results [18], for example, the unsupervised deep learning model [19] which incorporates synchrosqueezed wavelet transform, Fast Fourier Transform, and unsupervised deep Boltzmann machine; synchrosqueezed wavelet transform-fractality model [20]; and Fast Fourier Transform based wavelet finite element method [21].

In this study, we aim to combine active cable drive modes and Hilbert–Huang transform (HHT) method to monitor the health of suspension bridges’ hangers. We consider a suspension bridge equipped with a set of active cables/tendons which are used for vibration damping [22,23]. We propose to take advantage of the active cables drive modes to excite the suspension bridge, and use the HHT of the outputs to establish a damage detection index, based on the Hilbert marginal spectrum.

Indeed, the choice of HHT owes to its several merits demonstrated in many applications such as fatigue test of automobile gearbox [24,25], fault diagnosis in machinery [26,27] and locating combined damage in truss-type structures [28]. The HHT was proposed by Huang [29,30]. It has adaptive characteristics without any need to select a basic function, which may be advantageous for several applications. It mainly includes two aspects. First, the primary signals are decomposed into a series of time-dependent intrinsic mode functions (IMFs). Then, instantaneous frequency and amplitude are obtained and presented in the three-dimensional spectrum for every IMF, named as the Hilbert–Huang spectrum. The Hilbert marginal spectrum of the signal can be obtained by integrating the Hilbert–Huang spectrum along the time axis; it can accurately reflect the trend of the signal amplitude and the instantaneous frequency. Without any energy leakage, it has higher resolution and accuracy than the traditional Fourier spectrum [29].

The aim of this paper is to explore the potential of HHT as a method to establish a damage index for health monitoring of suspension bridges’ hangers. Since the method performs perfectly numerically, and for the sake of brevity, only the experimental implementation is presented. The paper is organized as follows: Section 2 presents the Hilbert marginal spectrum method for damage index estimation. Section 3 gives a brief overview of the bridge mock-up and describes its vibration characteristics in presence of active cables used for excitation. Section 4 mainly focuses on the experimental validation of the proposed method and discusses its shortcomings. Section 5 introduces a multiple active cable drive mode to improve the performance of the proposed method for cable damage detection. Finally, the findings and conclusions of this study are summarized at the end.

## 2. Theoretical Background

The classical vibration-based damage detection techniques have in common that they rely on modal parameters. For the past two decades, many effective modal estimation techniques have been used in the field of structural damage detection, such as Ibrahim time domain (ITD) method [31], eigensystem realization algorithm (ERA) [32], natural excitation technique (NExT) [33], stochastic subspace identification (SSI) [34] and the newly developed blind source separation (BSS) [35,36,37]. The accuracy of modal parameter estimation is determined by the existing test conditions and technology. Hence, the modal parameter-based damage detection method may be complex in most practical applications in civil engineering.

Alternatively, the HHT method can be directly used in damage detection and does not require knowing the modal parameters. For bridge-safety inspection, Huang [38] used a transient load to examine the nonlinear characteristics in a bridge vibration data to identify the damage. They used the HHT method to analyze the transient characteristic of the load and succeeded to locate the damaged spot.

In this study, we consider the structural loading generated by the active tendons, intended to be used for active vibration damping [22]; this is more convenient as it enables the use of the same excitation profile to study the healthy and the damaged structure. Hence, by simply comparing the Hilbert marginal spectrum of the damaged structure and the undamaged structure, it is straightforward to identify an efficient damage indicator.

### 2.1. Hilbert–Huang Transform (HHT)

Generally, a nonlinear and non-stationary complex signal is not suitable to be used to Hilbert transform. To use the HHT, the original signal should be decomposed into a number of intrinsic mode functions (IMFs) $c_{i}(t)$ (*i* = 1, 2, … *n*) and a residue $r_{n}\left(t\right)$. This process is known as empirical mode decomposition (EMD) [29].

(1) $$x\left(t\right)=\overset{n}{\underset{i=1}{\sum }}c_{i}\left(t\right)+r_{n}\left(t\right),$$

The IMF should be satisfied with the following two conditions [29]:

- In the whole data set, the number of extrema and the number of zero crossings must be equal or different at most by one.
- At any point, the mean value of the envelope defined by the local maxima and the envelope defined by the local minima is zero.

After using the Empirical Mode Decomposition (EMD) method, each intrinsic mode function component will satisfy the conditions of Hilbert transform. For each IMF component $c_{i}\left(t\right)$, the Hilbert transform is defined as:

(2) $$H\left[c_{i}\left(t\right)\right]=\frac{1}{\pi }P\int_{-\infty }^{\infty }\frac{c_{i}\left(\tau \right)}{t-\tau }d\tau ,$$

where *P* stands for the Cauchy principal value. This transform exists for all functions of class $L_{p}$ [25]. Using the Hilbert transform, the signal can be defined as:

(3) $$q_{i}\left(t\right)=c_{i}\left(t\right)+i\;H\left[c_{i}\left(t\right)\right]{=A}_{i}\left(t\right)e^{-i\theta (t)},$$

With the instantaneous amplitude:

(4) $$A_{i}\left(t\right)=\sqrt{c_{i}^{2}\left(t\right)+H{\left[c_{i}\left(t\right)\right]}^{2}},$$

The phase function:

(5) $$\theta_{i}\left(t\right)=\arctan\left(\frac{H\left[c_{i}\left(t\right)\right]}{c_{i}\left(t\right)}\right),$$

The instantaneous frequency is obtained by:

(6) $$\omega_{i}\left(t\right)=\frac{d\theta_{i}\left(t\right)}{dt},$$

After performing the Hilbert transform on each IMF component, the original data can be expressed as the real part in the following form:

(7) x(t)=Re{∑i=1nAi(t)ei∫ωi(t)dt},

Equation (7) gives both the amplitude and the frequency of each component as a function of time. The same data expanded in a Fourier representation would be:

(8) x(t)=Re[∑i=1nAieiωit],

where *A_i_* and *ω_i_* are constants. The contrast between Equations (7) and (8) is clear: the IMF represents a generalized Fourier expansion. The variable amplitude and the instantaneous frequency improve the efficiency of the expansion and enable the expansion to adapt to the nonlinear and non-stationary data. As the Hilbert transform on IMFs amplitude and frequency modulation are also clearly separated, the constant and fixed frequency limits of Fourier expansion have been overcome with variable amplitude and frequency representation [29]. This frequency–time distribution of the amplitude is referred to as the “Hilbert amplitude spectrum” H(ω,t), or simply “Hilbert spectrum”. With the Hilbert spectrum defined, the marginal spectrum, *h*(*ω*), is defined as:

(9) $$h(\omega )=\int_{0}^{T}H(\omega ,\;t)dt,$$

The marginal spectrum provides a measure of the total amplitude (or energy) contribution of each frequency value. The spectrum represents the cumulative amplitude of the whole data span in the probability sense. The Hilbert marginal spectrum can accurately represent the variation of the signal amplitude with the instantaneous frequency and has higher accuracy and resolution than the traditional FFT spectrum [29].

### 2.2. Damage Index

The data collected from damage location may contain important information about the damage in the structural member. The special features of this significant information are often reflected in the response amplitudes. In the instantaneous frequency range (*ω*_1_, *ω*_2_), the amplitude *A*, referred to as eigen amplitude, is defined as:

(10) $$A_{D}={\left[\int_{\omega_{1}}^{\omega_{2}}h(\omega )d\omega \right]}_{D},$$

(11) $$A_{H}={\left[\int_{\omega_{1}}^{\omega_{2}}h(\omega )d\omega \right]}_{H},$$

where *h*(*ω*) is the Hilbert marginal spectrum; *ω* is the instantaneous frequency and the instantaneous frequency range (*ω*_1_, *ω*_2_) is determined by the energy concentration range in the Hilbert marginal spectrum; and subscripts *H* and *D* stand for the healthy and damaged structure, respectively. It is worth mentioning that the input of the system should remain consistent in the test. In addition, the method assumes that the structure data (dynamics/signals) have been measured when it is healthy, where active cables are used for excitation. These data are then used as a reference.

The eigen amplitude reflects the amplitude distribution of the signal in the corresponding instantaneous frequency region. When a local damage occurs, it may lead to the change of the local physical parameters such as stiffness (and eventually mass), which in turn causes the change of the Hilbert marginal spectrum. The eigen amplitude in the same instantaneous frequency range also changes due to damage. Thus, by simply comparing the eigen amplitudes before and after damage, one can compute a damage index $DI_{v}$ of the structure by combining Equations (10) and (11); it is given by:

(12) $${DI}_{v}=\frac{\left|{\left[\int_{\omega_{1}}^{\omega_{2}}h(\omega )d\omega \right]}_{DV}-{\left[\int_{\omega_{1}}^{\omega_{2}}h(\omega )d\omega \right]}_{HV}\right|}{{\left[\int_{\omega_{1}}^{\omega_{2}}h(\omega )d\omega \right]}_{HV}},$$

where the subscript *v* stands for the vertical direction of the eigen amplitude (as the structure motion is dominant in this direction).

### 2.3. Proposed Methodology

Once the detection algorithm parameters are well defined (IMFs and the instantaneous frequency range), we calculate the damage index $DI_{v}$ as follow:

- First, the bridge (damaged and undamaged) is excited with the active cables and the response is measured at several measurement points. For the considered bridge, it is measured on the deck, near to the hangers’ attachment.
- Second, the data from the same measurement points (damaged and undamaged) are decomposed into *n*-empirical modes (IMFs). By analyzing the central frequencies of the IMFs, the related IMFs are selected which contain the information of the dominant modes (for the considered bridge, the first five vibration modes).
- Third, the Hilbert marginal spectrum of each measurement point is computed using the related IMFs (damaged and undamaged).
- Finally, the damage indices $DI_{v}$ are calculated using Equations (10)–(12) for each damage scenario.

**Figure 1:**
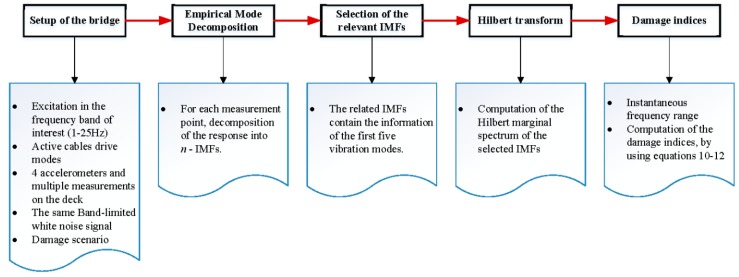
Damage detection procedure.

The flow chart of the proposal is shown in Figure 1.

## 3. Experimental Testing of the Suspension Bridge Mock-Up

### 3.1. Laboratory Suspension Bridge Model

**Figure 2:**
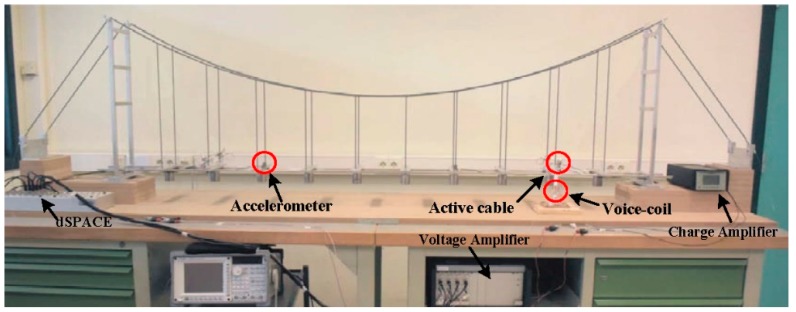
Laboratory mock-up equipped with four active cables connecting the pylon to the deck.

The laboratory mock-up of the suspension bridge is shown in Figure 2. It consists of two articulated towers of 0.62 m distant of 2.2 m; the deck is free to rotate at both ends and is attached to the catenary by two rows of 10 hangers.

The catenary consists of a steel cable with a diameter of 1 mm and the hangers are made of steel cables of 0.5 mm; the tension $T_{0}$ in the catenary and the hangers can be adjusted with screws; it is measured indirectly from the natural frequency f of the lateral modes of the cabes, according to the string formula:

(13) $$f=\frac{1}{2L}\sqrt{\frac{T_{0}}{\rho A}},$$

f being deduced from the lateral vibration of the cables, measured with a non-contact custom made laser sensor [39]. L is the length of the cable, ρ is its mass density and A is the cross-section. In this way, it was possible to distribute the tension in the hangers uniformly.

### 3.2. Active Cables

**Figure 3:**
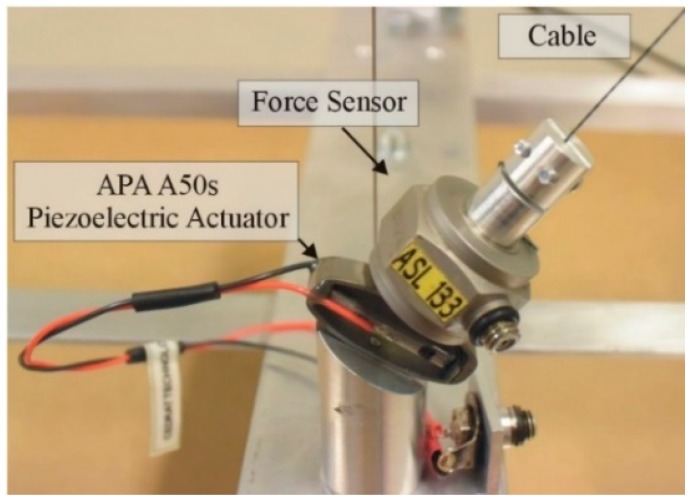
Detail of the active tendon.

Four active cables, with the same properties as the hangers, are added symmetrically to the mock-up in Figure 2. The chosen configuration is the one connecting the deck to the top of the pylons. The control authority of the active cables over the targeted modes depends directly on their attachment point on the deck. Thus, to maximize the control authority over several low frequency modes, and thus excite most of them, the active cables are attached on the deck near the second or the third hangers, as shown in Figure 2. The tension in the active cables is controlled with four APA 50 s piezoelectric actuators, from Cedrat technologies, collocated with force sensors (for active control purposes only), as shown in Figure 3.

**Figure 4:**
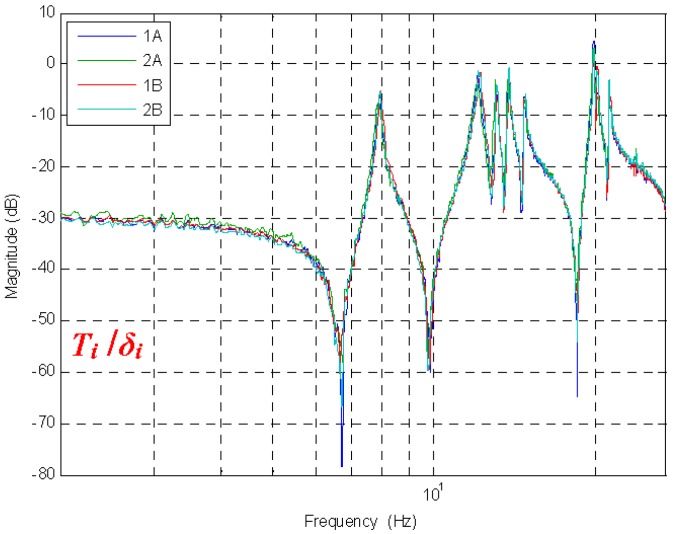
The transfer function of the four active cables $T_{i}/\delta_{i}$.

The transfer functions between the piezoelectric actuators ($\delta_{i}$) and their corresponding force sensors are shown in Figure 4. A band-limited white noise is used for excitation. Figure 4 contains important features of the bridge structure, namely, its symmetry and the tension in its cables. Indeed, such agreement between the transfer functions has been obtained after a complex tuning process of the bridge cables (the tension in the 2 catenaries, the 20 hangers, and the 4 active cables had to be tuned at the same time to reach a good symmetry).

A small magnet is attached to the deck and a voice coil is used to apply a disturbance force to the structure (only for modal identification, not for health monitoring) (Figure 2). The response of the deck is measured with four accelerometers, placed alternately along the deck. Due to the limitation of the hardware, the measurement is limited to four locations at the same time, by using four accelerometers. To cope with this limitation and obtain the response at the 20 locations, the sensors are moved along the bridge deck, where the same band-limited white noise signal is used for excitation.

**Figure 5:**
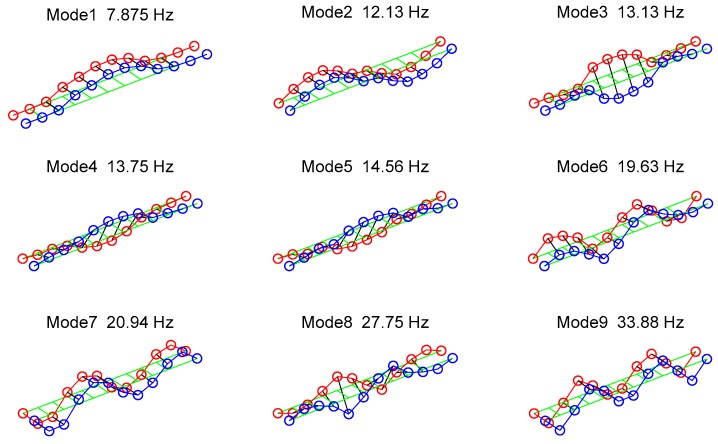
Experimental natural frequencies and mode shapes. The natural modal damping ranges from 0.1% to 0.8%.

Figure 5 shows the experimental natural frequencies and mode shapes extracted from the measured frequency response of the deck at 20 different points. The measured structural damping ratios range between 0.1% and 0.8%.

## 4. Experimental Implementation

**Figure 6:**
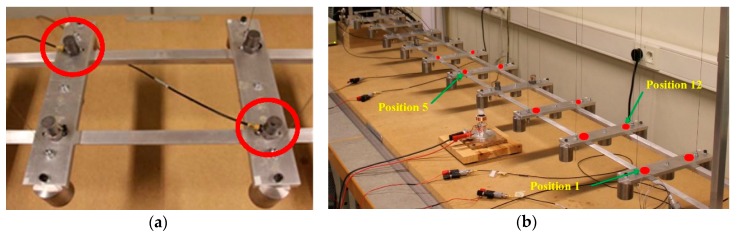
Layout of the accelerometers over the bridge deck (four accelerometers and multiple measurements): (**a**) the accelerometer; and (**b**) measuring position on the deck (one point near each hanger).

First, consider a single active cable to excite the structure (Cable 1A). The active cable is attached at close to the third hanger to ensure an effective excitation. The excitation consists of a band-limited white noise voltage applied to the piezoelectric actuator; a set of accelerometers monitor the deck acceleration in the vertical direction, which are laid as depicted in Figure 6.

The minimum length of time series *T*_min_ used for this experiment covers almost the whole dynamic behavior of the structure within the frequency band of interest [40,41], such that $T_{\min}=\frac{1000\sim 2000}{f_{\min}}$, where *f*_min_ is the lowest natural frequency in Hz (i.e., at least, the measurement must cover 1000–2000 oscillations associated with the first mode). Based on the preliminary FRF analysis, *f*_min_ is close to 8 Hz and *T*_min_ is selected as 250 s. To minimize the size of data, we selected a sampling rate of 1000 Hz (the highest resonance frequency is about 33 Hz). A low-pass analog filter at 100 Hz has been used to remove the high frequency noise and avoid any temporal aliasing.

**Figure 7:**
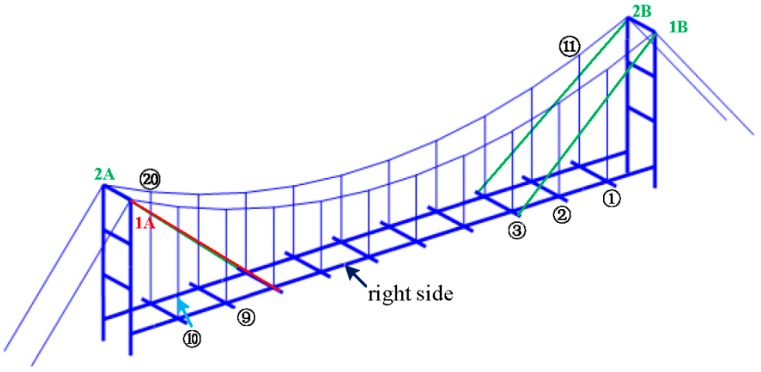
Cable number of mock-up. The hangers are numbered from 1 to 10 on one side of the deck, and from 11 to 20 on the other side. The active cables are 1A, 1B, 2A, and 2B.

Finally, the cables are numbered as shown in Figure 7: from 1 to 10 on one side, starting from the right, and from 11 to 20 on the opposite side. 1A, 2A, 1B, and 2B indicate the active cables.

### 4.1. Damage Detection with a Single Active Cable Drive: Scenarios A

Generally, damage leads to a decrease of the structural stiffness, thus damage in the bridge hangers is simulated by reducing their pre-stress. Based on Equation (13), the damage levels are simply tuned by properly tuning the resonance frequency of the cables. The implemented damage scenarios are described in Table 1. After each damage scenario, the tension in the cables is tuned back to its original value (healthy configuration), by identifying the resonance frequency of the lateral vibration using a custom-made sensor (Equation (13)).

#### 4.1.1. Intrinsic Mode Functions (IMFs)

**Figure 8:**
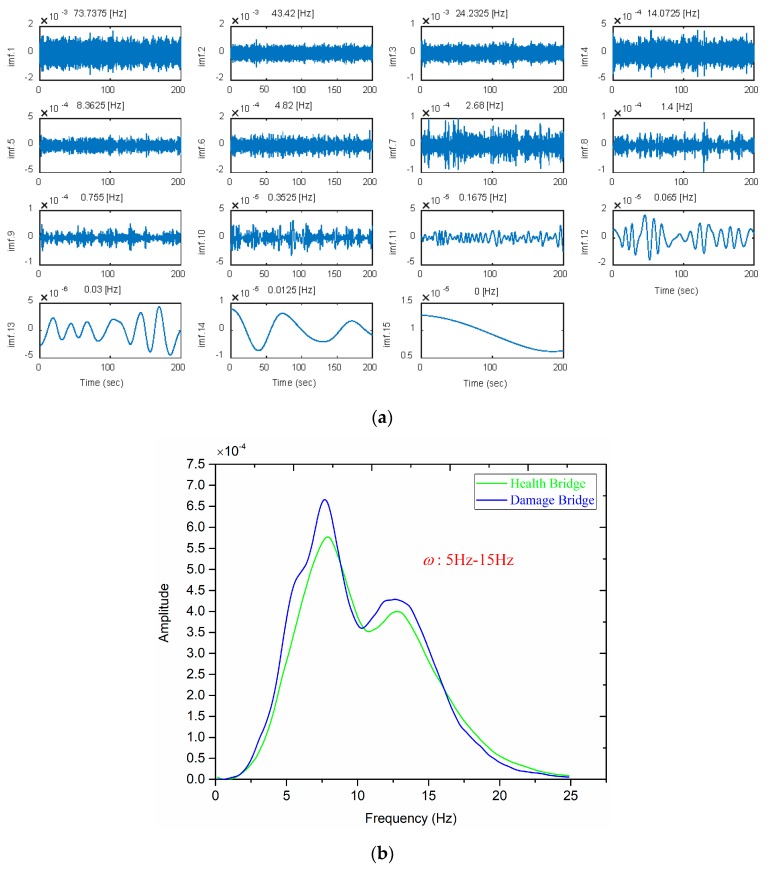
Results of HHT. The signal from the accelerometer at position 5 (Case A2): (**a**) IMFs in the time domain; and (**b**) Hilbert marginal spectrum.

To illustrate the process of the algorithm, consider the signal measured by the accelerometer located near the damaged Cable 5 (Case A2) as an example. Based on the empirical mode decomposition method (EMD), the signal is decomposed in several steps until (for this example, into 14 IMFs) a monotonous/constant residue is obtained (IMF 15). The detail process of EMD can found in [29]. For the considered example, the IMFs in the time domain are shown in Figure 8a. Each IMF contains different dynamic information of the structure and the central frequency is an effective way to evaluate the main component of each IMF. By analyzing the central frequencies of the IMFs (Figure 8a), observe that the main component of IMFs 4–6 contain the information of the first five vibration modes, which is also reflected in their Hilbert marginal spectrum, as shown in Figure 8b (although IMFs 3 and 7 count for a small contribution). These IMFs are chosen, as they exhibit a significant contribution of the structure modes, and the response of the structure is simply a combination of these modes (as the excitation is a white noise).

#### 4.1.2. Instantaneous Frequency Range (*ω*_1_, *ω*_2_)

To select a reasonable frequency range, we analyze the Hilbert marginal spectrums (Figure 8b); one can find that the energy is concentrated in the range of 5–15 Hz, which includes the first five vibration modes. Although the natural frequencies have a little sensitivity to the damage [42,43], Figure 8 indicates that the Hilbert marginal spectrum exhibits some obvious changes in the frequency range 5–15 Hz. Therefore, the frequency range (5 Hz, 15 Hz) is used in all the examples below. The calculation process of the damage index $DI_{v}$ is found in Section 2.3.

#### 4.1.3. Effect of Signal Error

**Figure 9:**
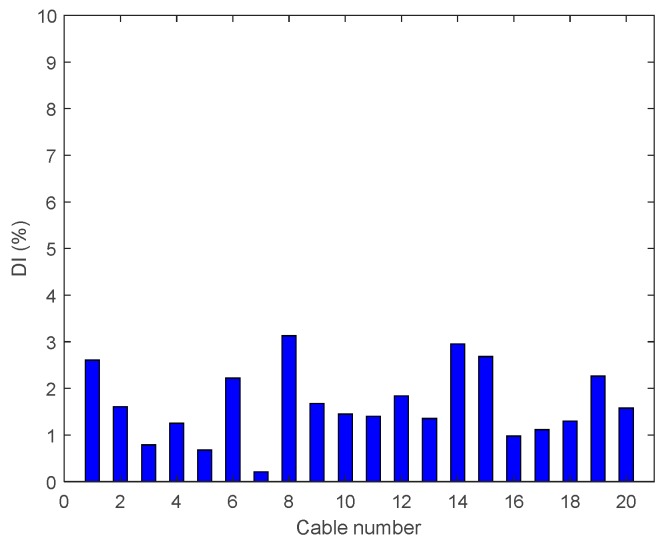
Effect of signal error.

The signal quality may lead to different damage detection results. To figure out the extent of the error (the process noise from the disturbances and the measurement noise from the sensors), we compare two samples of signals at a different time from the same position of the healthy bridge. The results are shown in Figure 9, where the maximum error is close to 3%. In a perfect configuration, without any noise, the damage indices would be zero. However, due to the measurement noise and the different excitations, small levels of damage indices are extracted (less than 3%); this can be considered as the noise on the damage index.

#### 4.1.4. Results and Discussion

• Single Damage Scenario A: Case A1 and Case A2

**Figure 10:**
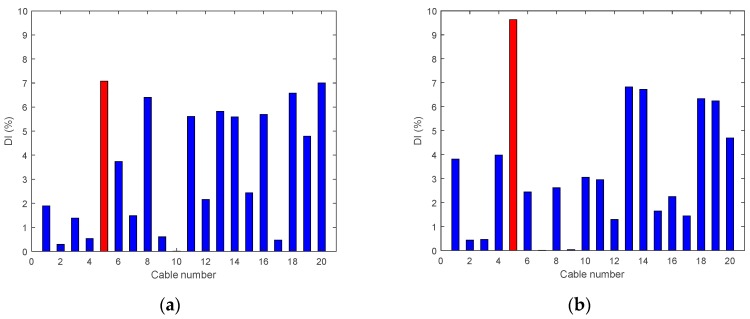
Damage indices for a damage at Cable 5: (**a**) Case A1 (50% reduction of tension); and (**b**) Case A2 (95% reduction of tension).

The first two damage cases assume Cable 5 is damaged. The results of the damage indices are shown in Figure 10, for both cases. In Figure 10a, the damage index $DI_{v}$ refers to the various positions of the accelerometers along the deck; it is maximum for the accelerometer located at the position of the damaged hanger. However, Number 18 and Number 20 have similar levels too, which may lead to confusion. In Figure 10b, where the damage level is about 95%, the $DI_{v}$ shows a maximum value at the damaged location. In this damage case, the vertical damage index is able to detect 95% of damage to the cable successfully at the center of the bridge span and confirm the actual location of the damage.

• Single Damage Scenario A: Case A3 and Case A4

**Figure 11:**
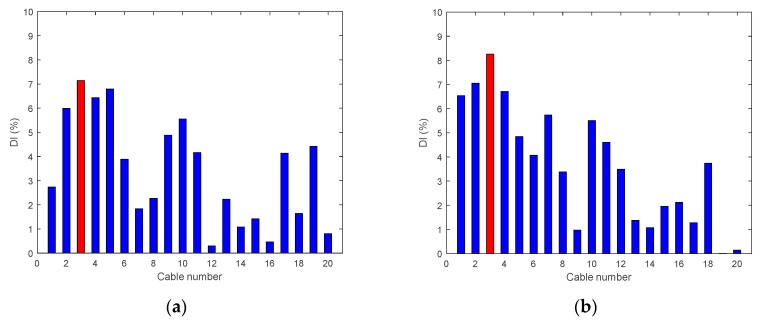
Damage indices for damage Cable 3: (**a**) Case A3 (50% reduction of tension); and (**b**) Case A4 (95% reduction of tension).

Figure 11 illustrates the $DI_{v}$ for Cases A3 and A4, where the tension of Cable 3 has been reduced. It can be seen that $DI_{v}$ peaks at the exact damage location for Case A4, but less precisely for Case A3.

• Single Damage Scenario A: Case A5

**Figure 12:**
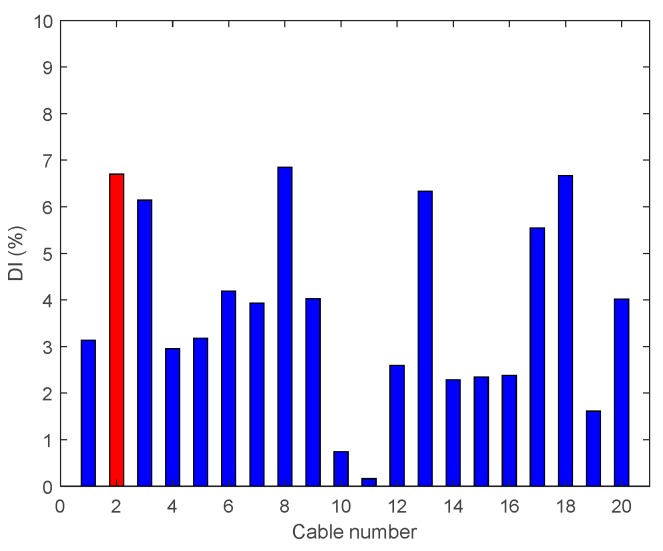
Damage indices for 95% damage Cable 2.

In Case A5, the tension of Cable 2 is reduced by 95%. Contrary to expectation, we do not find a significant peak in Figure 12 and the vertical eigen amplitude method with a single active cable drive fails to detect the 95% damage. This can be associated with the fact that Cable 2 is located near the edge of the bridge, and, by observing the mode shapes in Figure 5, we can see that the edge of the deck has smaller modal contribution compared to the mid-span (for most of the excited modes), which explains the insensitivity of the method to the damage of Cable 2. Thus, to detect the damage in Cable 2, one needs to excite the vibration modes where the cable has a higher modal contribution. This can be performed by using several active cables, as described further in Section 4.2.

• Multiple Damage Scenario A: Case A6

**Figure 13:**
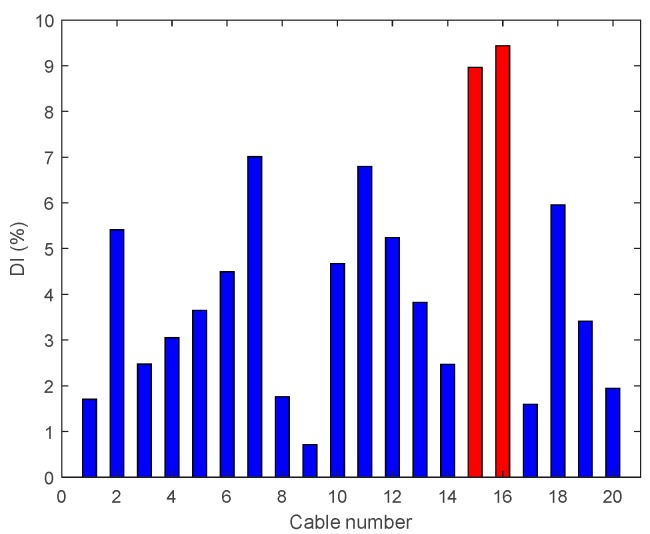
Damage indices for 95% Damage Case A6.

In this damage case, a tension reduction of 95% is made at two cables located at mid-span (Cables 15 and 16). The results are shown in Figure 13 and it can be seen that $DI_{v}$ locates precisely the damage.

• Multiple Damage Scenario A: Case A7

**Figure 14:**
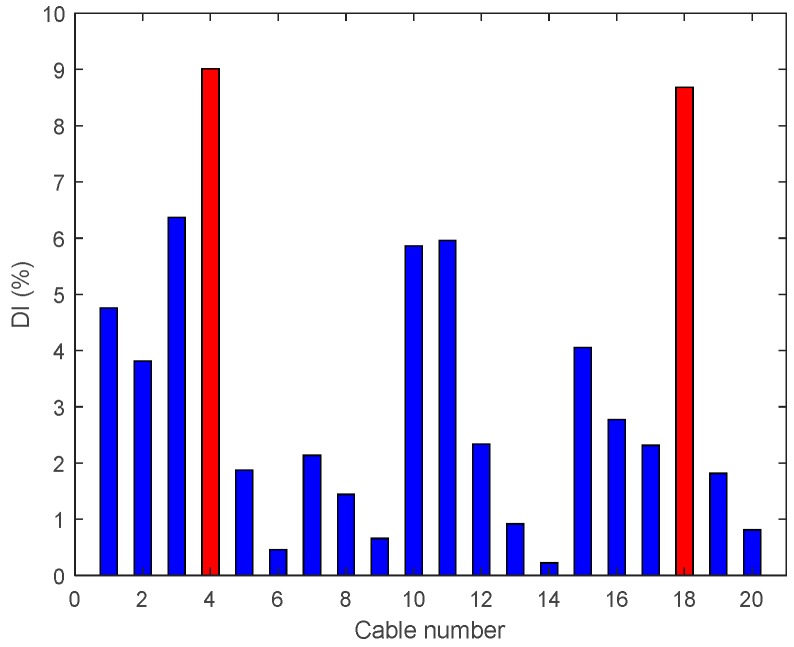
Damage indices for 95% Damage Case A7.

In Damage Case A7, there are two 95% tension reductions at two cables near the middle and third span (Cables 4 and 18). The results are shown in Figure 14 and it is evident that $DI_{v}$ shows two peaks corresponding to the two damage locations.

• Multiple Damage Scenario A: Case A8

**Figure 15:**
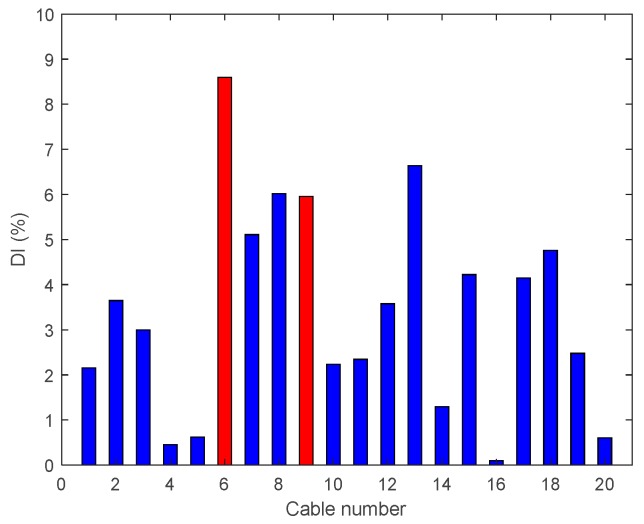
Damage indices for 95% Damage Case A8.

The last damage case studied under the multiple damage scenarios are with 95% tension reductions at the middle and quarter span (Cables 6 and 9); the results are shown in Figure 15. It is evident that $DI_{v}$ is able to locate the damage in the middle span but gives an incorrect prediction in the quarter span of the cable near the edge of the bridge.

The foregoing results indicate that $DI_{v}$ is appropriate to locate multiple damages provided the damage location is located near an element whose contribution to the excited modes is significant. Moreover, it has been shown that damages located near the deck supports are difficult to detect. This situation is further analyzed below.

### 4.2. Damage Detection with Multiple Active Cables Drive: Scenarios B

**Figure 16:**
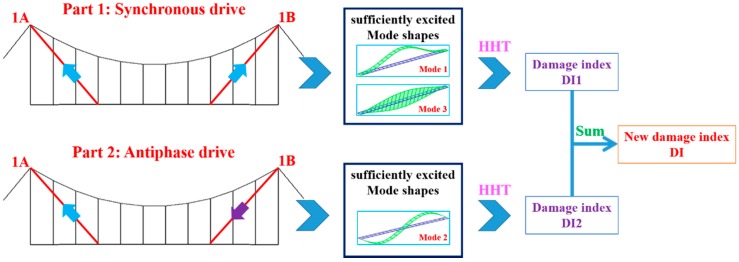
Multiple active cables drive process.

Due to the low modal contribution of some modes when the structure is excited with a single active cable, the $DI_{v}$ is unable to detect the damage in the cables located near the edge of the bridge. To deal with such a problem, we propose to use two active cables for exciting the deck instead of one single cable (it is also possible to excite with four active cables). This enables the possibility of exciting the structure with a spatial forcing shape similar to the mode shapes of interest, and thus excite efficiently the modes with high modal amplitude near the clamp. Two active cables (1A and 1B) are used to drive the mock-up. The whole drive process is divided into two parts: synchronous drive, where the two cables are driven with the same signal (e.g., more suitable for exciting Mode 1), and the antiphase drive, where the two cables are driven with opposite sign signals, as shown in Figure 16. We use the same band-limited white noise as before.

As shown in Figure 16, Modes 1 and 3 tend to be well excited by a synchronous drive of Cables 1A and 1B, while the antiphase drive tends to excite Mode 2. By using the two drive modes alternatively, a new enhanced Damage Index can be defined as the sum of the two damage indices $DI_{1}$ and $DI_{2}$ associated to each drive mode. Table 2 shows the details of the various damage scenarios considered with multiple active cables drive.

• Single Damage Scenario B: Case B1, B2, and B3

**Figure 17:**
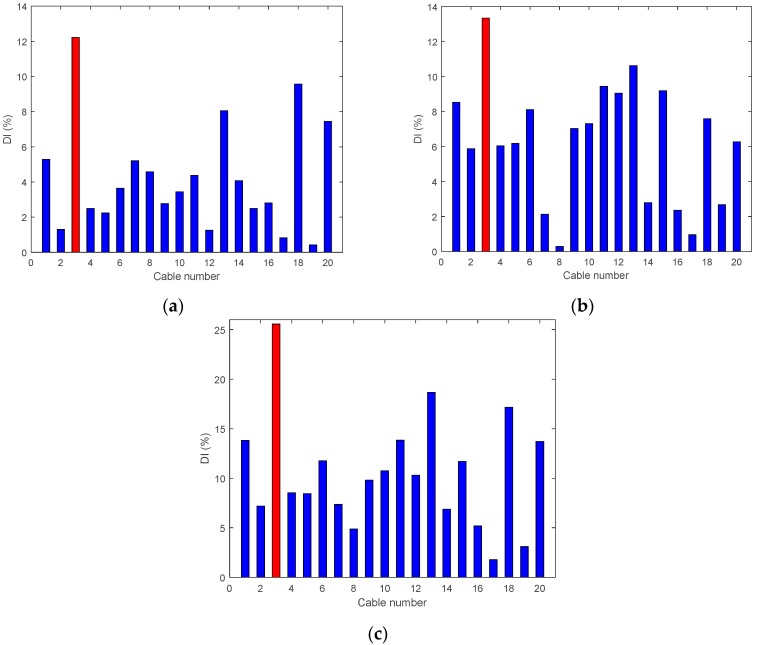
Enhanced damage indices for 95% Damage Case B1: (**a**) results of the synchronous drive; (**b**) results of the antiphase drive; and (**c**) results of synchronous drive + antiphase drive.

**Figure 18:**
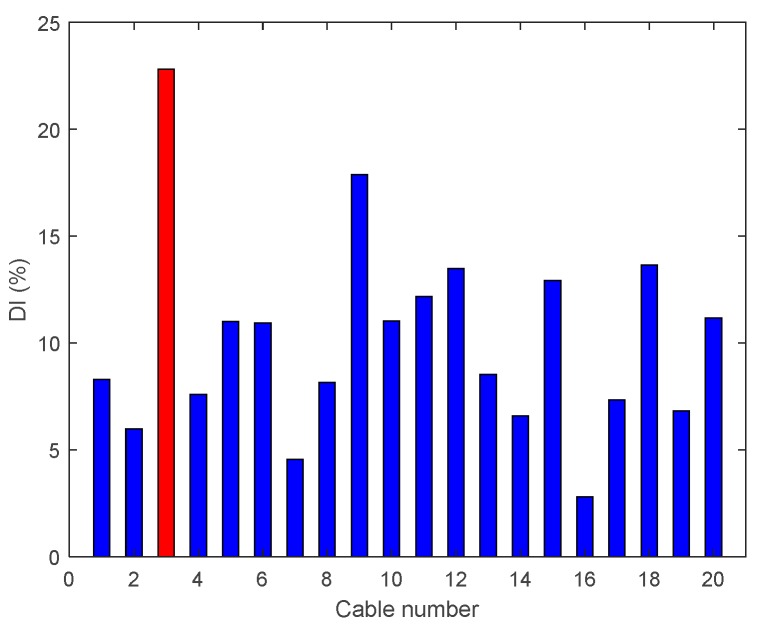
Enhanced damage indices for 80% Damage Case B2.

**Figure 19:**
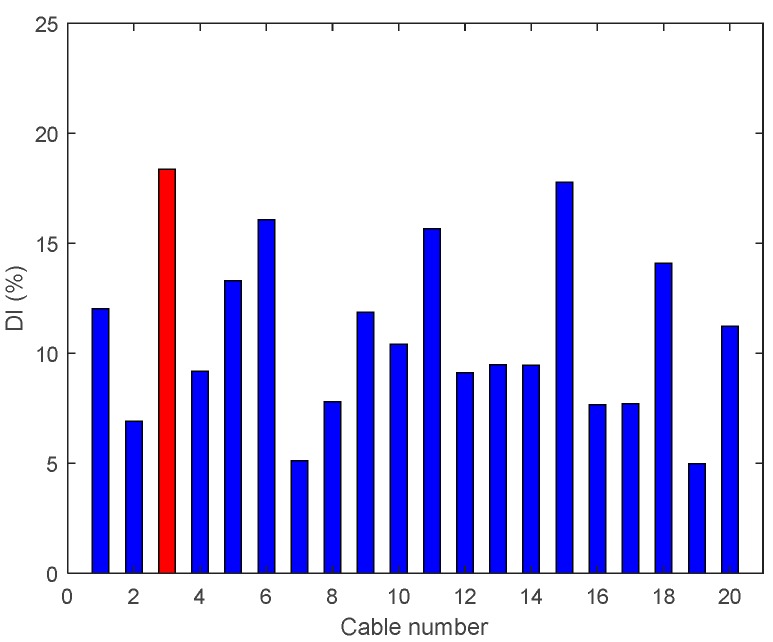
Enhanced damage indices for 50% Damage Case B3.

In these three damage cases, 95%, 80%, and 50% tension reductions are made at the cable located at the third span (Cable 3) and the results are shown in Figure 17, Figure 18 and Figure 19.

Figure 17 shows the results of each step in the damage detection process for Case B1. Compared with the results of Case A4, the detection capability has been significantly improved. In Figure 18, the enhanced $DI_{v}$ shows an evident peak at the damage location. Under this hybrid drive, the enhanced damage index becomes more sensitive to lower damage levels located near the clamp. However, the enhanced damage index still fails to detect accurately a damage with low severity of 50%, as shown in Figure 19.

• Single Damage Scenario B: Case B4 and B5

**Figure 20:**
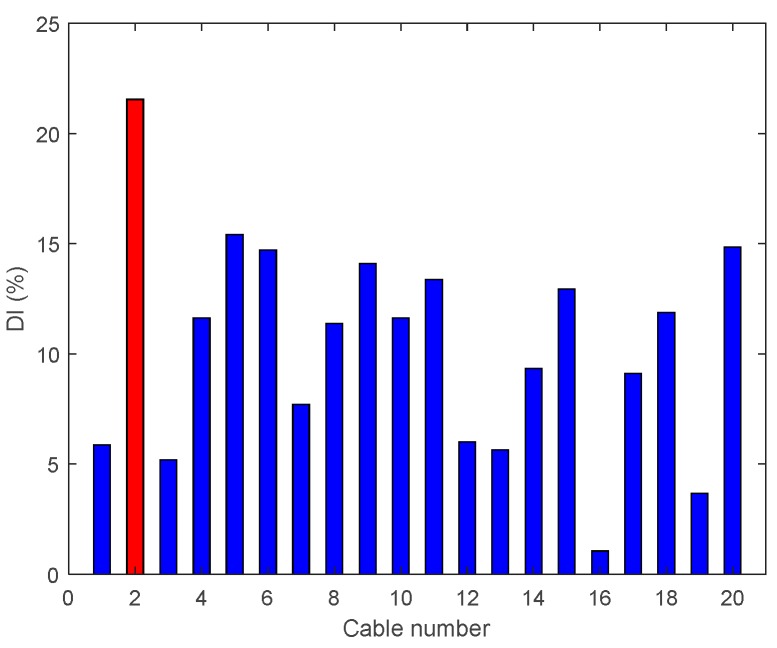
Enhanced damage indices for 90% Damage Case B4.

**Figure 21:**
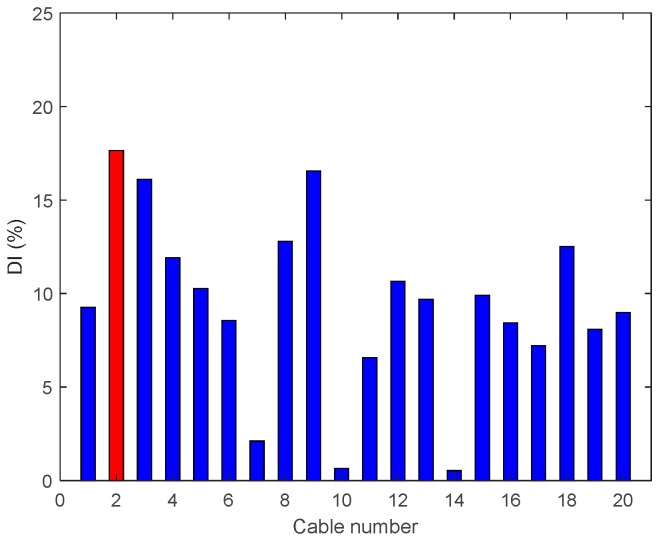
Enhanced damage indices for 80% Damage Case B5.

Figure 20 shows a significant outcome of Case B4, from which it is evident that the enhanced damage index $DI_{v}$ gives a correct prediction of the damage location. This result could not be achieved with a single active cable drive (Damage Case A5). However, once the damage intensity reduces to 80%, the enhanced $DI_{v}$ is unable to locate the damage, as shown in Figure 21.

• Multiple Damage Scenario B: Case B6

**Figure 22:**
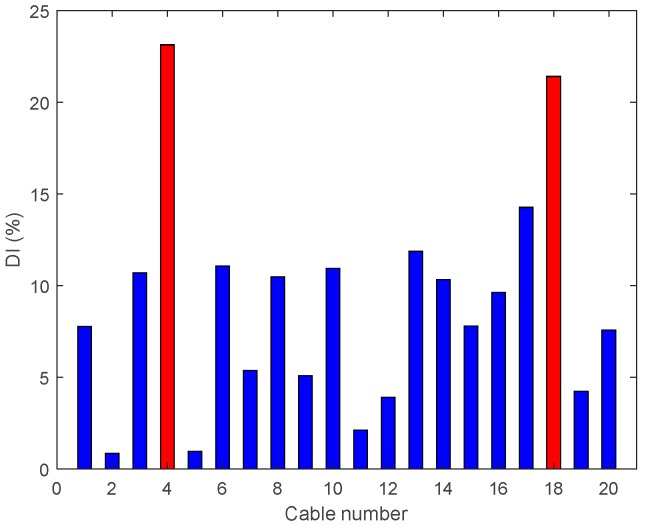
Enhanced damage indices for 80% Damage Case B6.

Figure 22 shows the enhanced $DI_{v}$, where two cables have seen their tension reduced by 80%. The enhanced $DI_{v}$ locates precisely the damaged cables.

• Multiple Damage Scenario B: Case B7

**Figure 23:**
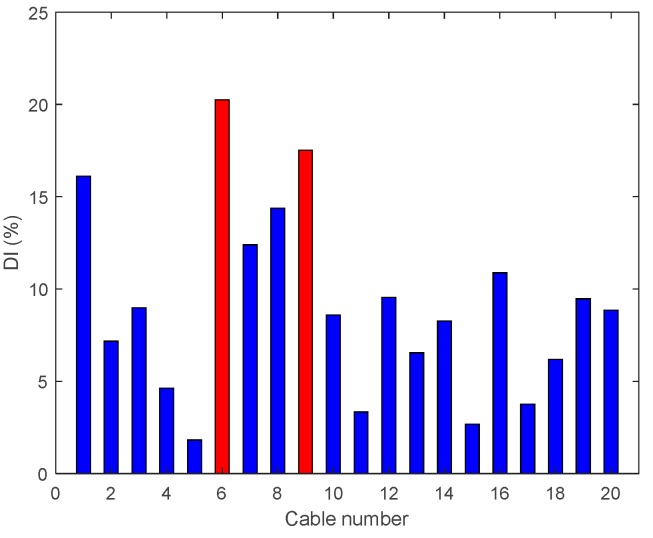
Enhanced damage indices for 80% Damage Case B7.

This damage case also considers multiple damage scenarios with 80% tension reduction of the cables located in the middle and near the quarter span (Cables 6 and 9); the results are shown in Figure 23. The $DI_{v}$ is able to locate the damage in the middle span of the Cable 6 but gives an incorrect prediction at the quarter span of Cable 9, located near the edge of the bridge (exhibiting a small modal amplitude).

### 4.3. Comparison with Traditional Methods

In this section, the traditional modal parameters-based damage detection methods: Coordinate Modal Assurance Criterion (COMAC) [44], Enhanced Coordinate Modal Assurance Criterion (ECOMAC) [45], Mode Shape Curvature (MSC) [46], Modal Strain Energy (MSE) [47] and Modal Flexibility (MF) [48] are introduced to detect the damage in the bridge. Only few scenarios are considered. The experimental results are summarized in Table 3. These results are extracted from a separate study under consideration [49].

As one observes in Table 3, none of the traditional methods can detect the low-level damages, corresponding to a loss of 80% of the hanger’s tension (Cases B2, B6 and B7). When the damage intensity is high, up to 95% tension loss, two traditional methods (MSE and MF) are able to detect the damage successfully and confirm the actual location of the damage (Case B1). However, by simply observing these results (Table 3), one can find that, once the damage intensity reduces to less than 90% and the damage position deviates to the edge of deck, only the MF method locates precisely the damaged cable (Case B4). For the 80% damage level near the mid-span (Cases B2 and B6), the performance of the enhanced $DI_{v}$ is superior to the traditional methods.

## 5. Conclusions

In this paper, we investigate and validate experimentally the possibility of using active cables to excite the structure to detect and locate damages in the hangers of a suspension bridge. The proposed method, based on the Hilbert marginal spectrum, has been successfully implemented and validated through an extensive set of damage scenarios. The damage index has been found very effective for single and multiple damages provided the damage location is sufficiently excited. The sensitivity of the damage index has been enhanced by using several cables in multiple driving modes.

Based on the presented results and interpretations, the main findings are summarized as follows:

- The active cable drive is an effective way to excite the suspension bridge. Combined with Hilbert–Huang transform (HHT), a damage index ($DI_{v}$) is constructed for damage detection of suspension bridge hangers. The proposed damage index ($DI_{v}$) showed promising damage detection capability.
- The hybrid drive with multiple active cables can improve significantly the damage detection capability and is able to detect single and multiples damages with small modal amplitudes. For the 80% damage level near the mid-span, the performance of the enhanced $DI_{v}$ is superior to the traditional modal parameters-based damage detection methods (COMAC, ECOMAC, MSC, MSE and MF). However, for some configurations, the enhanced damage index fails to detect damages, mainly for low-level damage.
- This paper validates the feasibility of the proposed method under laboratory conditions; however, some limitations are inevitable. Indeed, reducing the tension in the hangers is not representative of damage as it modifies only the lateral stiffness of the cable and does not affect its longitudinal stiffness, which significantly contributes to the deck vertical stiffness. Thus, a more representative damage scenario must be used to conclude on the quality of the algorithm (e.g., cutting slightly the cable).
- Finally, in this paper, we consider the influence of noise and ignored the influence of environmental and operational variability on the dynamic behavior. It is well known that suspension bridges are insensitive to tiny damages. Thus, a small damage intensity would be detectable by this method (equivalent to 80% reduction of the hanger tension), which is a satisfactory result. Nevertheless, to confirm these findings, the extension to field experiments under realistic conditions should be further investigated. More importantly, some quantitative results can be obtained. A potential test case would be the Seriate footbridge, located in Bergamo, Italy, and considered as a test case in a previous study [22].

## Figures and Tables

**Table 1:** Single cable drive: damage scenarios A.

Damage Case	Location	Severity of Damage (Tension Reduction)
Single damage scenario		
Case A1	Cable 5	50%
Case A2	Cable 5	95%
Case A3	Cable 3	50%
Case A4	Cable 3	95%
Case A5	Cable 2	95%
Multiple damage scenario		
Case A6	Cable 15 and Cable 16	95%
Case A7	Cable 4 and Cable 18	95%
Case A8	Cable 6 and Cable 9	95%

**Table 2:** Damage scenarios B.

Damage Case	Location	Severity of Damage (Tension Reduction)
Single damage scenario		
Case B1	Cable 3	95%
Case B2	Cable 3	80%
Case B3	Cable 3	50%
Case B4	Cable 2	90%
Case B5	Cable 2	80%
Multiple damage scenario		
Case B6	Cable 4 and Cable 18	80%
Case B7	Cable 6 and Cable 9	80%

**Table 3:** Comparison of damage detection methods.

Case #	COMAC	ECOMAC	MSC	MSE	MF	Enhanced *DI_v_*
B1	X	X	OO	O	O	O
B2	X	X	OO	OO	OO	O
B4	X	X	X	X	O	O
B6	X	OO	X	OO	OO	O
B7	X	OO	OO	OO	OO	OO

O, damage detected and located; OO, damage detected but not located; X, damage not detected.
